# Scribner’s Illustrated Monthly Magazine

**Published:** 1875-06

**Authors:** 


*Scribner's Illustrated Monthly Magazine*

Is one of the purest and best of our literary monthlies. Under the guiding hand of Dr. Holland, it has achieved a success its merits so richly deserve. Dr. Holland (an M. D.,) has a story of very great beauty and moral strength running through its numbers. "Seven Oaks" is as beautiful in finish, as touching in pathos, and as thrilling in incidents, as "Author Bonicastle." What we specially admire is the deep religious tone of Dr. Holland's writings. Our friends will not be disappointed in Scribner.

